# Experimental demonstrations of unconditional security in a purely classical regime

**DOI:** 10.1038/s41598-021-83724-w

**Published:** 2021-02-18

**Authors:** Byoung S. Ham

**Affiliations:** Center for Photon Information Processing, School of Electrical Engineering and Computer Science, Gwangju Institute of Science and Technology, 123 Chumdangwagi-ro, Buk-gu, Gwangju, 61005 South Korea

**Keywords:** Quantum information, Quantum optics

## Abstract

So far, unconditional security in key distribution processes has been confined to quantum key distribution (QKD) protocols based on the no-cloning theorem of nonorthogonal bases. Recently, a completely different approach, the unconditionally secured classical key distribution (USCKD), has been proposed for unconditional security in the purely classical regime. Unlike QKD, both classical channels and orthogonal bases are key ingredients in USCKD, where unconditional security is provided by deterministic randomness via path superposition-based reversible unitary transformations in a coupled Mach–Zehnder interferometer. Here, the first experimental demonstration of the USCKD protocol is presented.

## Introduction

Quantum key distribution (QKD) has been intensively researched for unconditionally secured key distribution over the last several decades^1–13^. Since the first QKD protocol of BB84^1^, various types of QKD protocols have been successfully demonstrated using optical fibers, free space, and even satellites^10^. Regardless of QKD type, the essential requirements for unconditional security are lossless quantum channels and perfect single-photon detectors. Moreover, a deterministic nonclassical light source is required for potential applications of QKD such as online banking and quantum internet. So far, none of these requirements have been fully satisfied. As a result, the unconditional security of QKD lied in the no-cloning theorem based on Heisenberg’s uncertainty principle^14^ cannot be fulfilled unless quantum loopholes are completely closed^6–13^. The bedrock of no-cloning theorem for the unconditional security in QKD is the quantum superposition between binary bases, resulting in eavesdropping randomness^14^. To initiate quantum superposition-based unconditional security in QKD, the basis of keys cannot be orthogonal. This is the fundamental difference of QKD compared with classical cryptography based on orthogonal bases.

Recently, a completely different protocol for unconditionally secured classical key distribution (USCKD) has been proposed to overcome the limitations of QKD mentioned above as well as to understand the basic quantum features in a classical regime^15^. Compared with quantum superposition-caused randomness in QKD, USCKD achieves unconditional security via path superposition in a Mach–Zehnder interferometer (MZI), where a coupling method between two MZIs plays a key role^16^. Unlike QKD, USCKD is based on a purely classical system of MZIs with orthogonal bases. Thus, USCKD seems to be self-contradicting because the unconditional security of QKD is based on non-orthogonal bases. Here, secrete of unconditional security in USCKD is in the path superposition-caused measurement randomness between orthogonal bases. According to information theory, randomness represents that there is no information to eavesdrop^17^. Moreover, USCKD results in key distribution determinacy between two remote parties via the coherence physics of MZI, even without post-measurement of sifting in QKD. The key distribution determinacy in USCKD is provided by reversible unitary transformations such as in quantum optical memories^18,19^. Thus, two-way communication channels are adapted to provide eavesdropping randomness and directional determinacy to form coupled MZI channels^15^.

The fundamental physics of USCKD has been studied in a coupled MZI system^15^, where a specific phase relationship between the coupled MZIs results in nonclassical features of coherence de Broglie wavelength (CBW)^16,20^. CBW is a classical version of photonic de Broglie wavelength (PBW), where PBW is a typical macroscopic quantum feature studied for quantum sensing and quantum metrology over the last few decades^21–27^. Recently, experimental demonstrations of CBW have been successfully performed, where USCKD represents a special state of CBW under the same physics^28^. Thus, CBW as well as USCKD have been understood as a macroscopic feature^15,16^. In that sense, conventional understanding of the quantum nature limited to the microscopic world satisfying the uncertainty principle has been intrigued and expanded toward the macroscopic world, such as in the case of Schrodinger’s cat^29,30^. Here, USCKD is experimentally demonstrated for the proof of principle of unconditional security in a purely classical regime of coupled MZIs. This study may open the door to coherence quantum technology, overcoming limitations in conventional quantum technologies confined to the microscopic world^1–13,21–27^.

## Results

**Figure 1:**
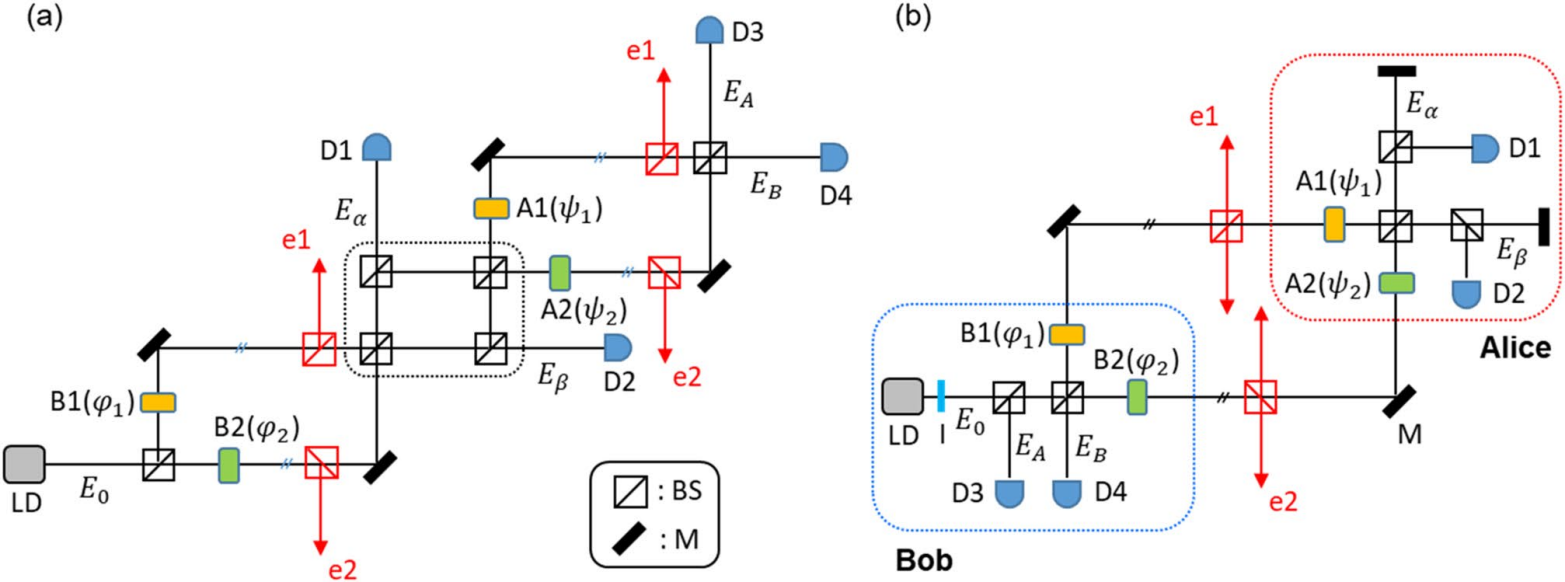
A schematic of unfolded USCKD for (**a**) unfolded and (**b**) folded configurations. Aj($\psi_{j}$) and Bj($\varphi_{j}$) represent an acousto-optic modulator j for Alice and Bob with phase basis ${\uppsi } \in \left\{ {0,\pi } \right\}$ and $\varphi \in \left\{ {0,\pi } \right\}$, respectively, where ${\uppsi } = \psi_{12} \left( { = \psi_{1} - \psi_{2} } \right)$ and ${{\varphi }} = \varphi_{12} \left( { = \varphi_{1} - \varphi_{2} } \right)$. The e1 and e2 in red denote eavesdropping paths by Eve. LD: Laser, I: isolator, BS: unpolarizing beam splitter, M: mirror, and Dj: detector j. All Aj’s are synchronized via microwave generators at 80 MHz.

Figure 1a shows an unfolded scheme of USCKD^15^ based on orthogonal bases of coherent light for classical key distribution, where two MZIs are coupled symmetrically with $\varphi_{12} = \psi_{12}$ and $\zeta_{ij} = \zeta_{i} - \zeta_{j}$. This means that the basic scheme of USCKD is composed of two identical MZIs via quantum superposition (see the dotted box) between them. Here, the coupling method for superposition plays an important role^15,16^. Unlike the symmetric coupling for USCKD in Fig. 1, CBW is based on asymmetric coupling, in which the asymmetry represents a ${\uppi } -$ phase shift to the second MZI of ${\psi s}$^16^. In each MZI, two phase bases $\left( {0, \pi } \right)$ of each path can be controlled by an acousto-optic modulator (AOM) pair, in which each AOM driving frequency plays a key role for the phase control of the MZI (discussed in experiments). In Fig. 1a, the $\varphi_{j} -$ based first MZI belongs to Bob for key preparation, while the $\psi_{j} -$ based second MZI belongs to Alice to set the key. When Fig. 1a is folded for a round trip USCKD configuration, the right-end BS meets the left-end BS, resulting in Fig. 1b. In other words, the detectors D3 and D4 with phase shifters B1 and B2 belong to Bob, while D1 and D2 with A1 and A2 phase shifters belong to Alice. Alice and Bob have a basis set, ${\uppsi } \in \left\{ {0,\pi } \right\}$ and ${{\varphi }} \in \left\{ {0,\pi } \right\}$, respectively, where ${\uppsi } \equiv \psi_{12}$ and ${{\varphi }} \equiv \varphi_{12}$.

For the experiments, all AOMs are set to be in-phase, and the phase control of the MZI system relies only on the upper AOM A1 via a two-channel function generator (AFG3102, Tektronix). For this, the lower AOM driving frequencies are fixed at 80 MHz sharp. To select a phase basis for each optical key, all four AOMs are synchronized to driving frequency generators, PTS160, PTS250, and AFG3102. The lower two AOMs, B2 and A2, are controlled by PTS160 and PTS250, respectively. The upper two AOMs, B1 and A1, are controlled by AFG3102. Thus, there are four possible phase basis combinations (see "Theory").

A sophisticated eavesdropper Eve attacks the transmission lines in both MZI channels via BSs, as shown by the red lines (e1 and e2), to form the same interferometric scheme as Alice’s or Bob’s. Unlike QKD, such a channel attack is allowed in USCKD without revealing her existence to Bob and Alice. Due to measurement randomness or indistinguishability in MZI, however, Eve’s chance to extract the correct phase information is 50% on average, resulting in unconditional security^15^. This randomness of 50% is the bedrock of unconditional security in USCKD. Here, it should be noted that Eve cannot distinguish the set basis by the MZI channel attack due to path superposition unless the set basis is known (see section A of the Supplementary Information). Regarding classical cryptographic researches, fundamental primitives for security have been developed to protect public key encryption, digital signature, or tag encoding schemes in networks from side-channel attack such as related-key attacks^31^ or network pollution attacks^32^. Thus, the present research of USCKD ensures those classical security systems.

### Theory

For an analytic approach, the matrix representation for the first MZI in Fig. 1a is given:

1 $$\left[ {\begin{array}{*{20}c} {E_{\alpha } } \\ {E_{\beta } } \\ \end{array} } \right] = \left[ {BS} \right]\left[ \varphi \right]\left[ {BS} \right]\left[ {\begin{array}{*{20}c} {E_{0} } \\ 0 \\ \end{array} } \right] = \frac{1}{2}\left[ {\begin{array}{*{20}c} {1 - e^{i\varphi } } & {i\left( {1 + e^{i\varphi } } \right)} \\ {i\left( {1 + e^{i\varphi } } \right)} & { - \left( {1 - e^{i\varphi } } \right)} \\ \end{array} } \right]\left[ {\begin{array}{*{20}c} {E_{0} } \\ 0 \\ \end{array} } \right],$$

where ${{\varphi }} = \varphi_{12}$ and $E_{0}$ is the input field of coherent light from LD. The BS matrix is $$\left[ {BS} \right] = \frac{1}{\sqrt 2 }\left[ {\begin{array}{*{20}c} 1 & i \\ i & 1 \\ \end{array} } \right]$$, and the matrix of a phase shifter between two MZI paths is $$\left[ \varphi \right] = \left[ {\begin{array}{*{20}c} 1 & 0 \\ 0 & {e^{i\varphi } } \\ \end{array} } \right]$$. Thus, the corresponding output intensities detected by D1 and D2 are as follows, respectively:

2 $$I_{\alpha } = \frac{1}{2}\left( {1 - cos\varphi } \right),$$

3 $$I_{\beta } = \frac{1}{2}\left( {1 + cos\varphi } \right),$$

where $I_{j} = E_{j} E_{j}^{*}$. Depending on the orthogonal phase basis of ${{\varphi }} \in \left\{ {0,\pi } \right\}$ in MZI, the output field intensity becomes either $I_{\alpha }$ or $I_{\beta }$. Thus, Alice knows what basis is chosen by Bob by her visibility ($V_{\alpha \beta } )$ measurements (see section B of the Supplementary Information)^15^. This represents the MZI propagation directionality. Here, it should be noted that the phase basis selection in ${{\varphi }}$ $\left( \psi \right)$ belongs to Bob (Alice) for key preparation (confirmation) according to the USCKD protocol^15^. The output field from the first MZI is inserted into the second MZI via symmetric superposition (dotted box) for Alice’s control. From the second MZI, the final output fields $E_{A}$ and $E_{B}$ are obtained as:

4 $$\begin{aligned} \left[ {\begin{array}{*{20}c} {E_{A} } \\ {E_{B} } \\ \end{array} } \right] & = \left[ {BS} \right]\left[ \psi \right]\left[ {BS} \right]\left[ {\begin{array}{*{20}c} {E_{\alpha } } \\ {E_{\beta } } \\ \end{array} } \right], \\ & = - \frac{1}{2}\left[ {\begin{array}{*{20}c} {e^{i\varphi } + e^{i\psi } } & { - i\left( {e^{i\varphi } - e^{i\psi } } \right)} \\ {i\left( {e^{i\varphi } - e^{i\psi } } \right)} & {e^{i\varphi } + e^{i\psi } } \\ \end{array} } \right]\left[ {\begin{array}{*{20}c} {E_{0} } \\ 0 \\ \end{array} } \right]. \\ \end{aligned}$$

Owing to the binary phase bases of ${{\varphi }}$ and ${\uppsi }$, there are four combinations of phase bases between Bob and Alice for the key distribution:

(i) $$\varphi = 0$$; $${\uppsi } = 0$$

**Figure 2:**
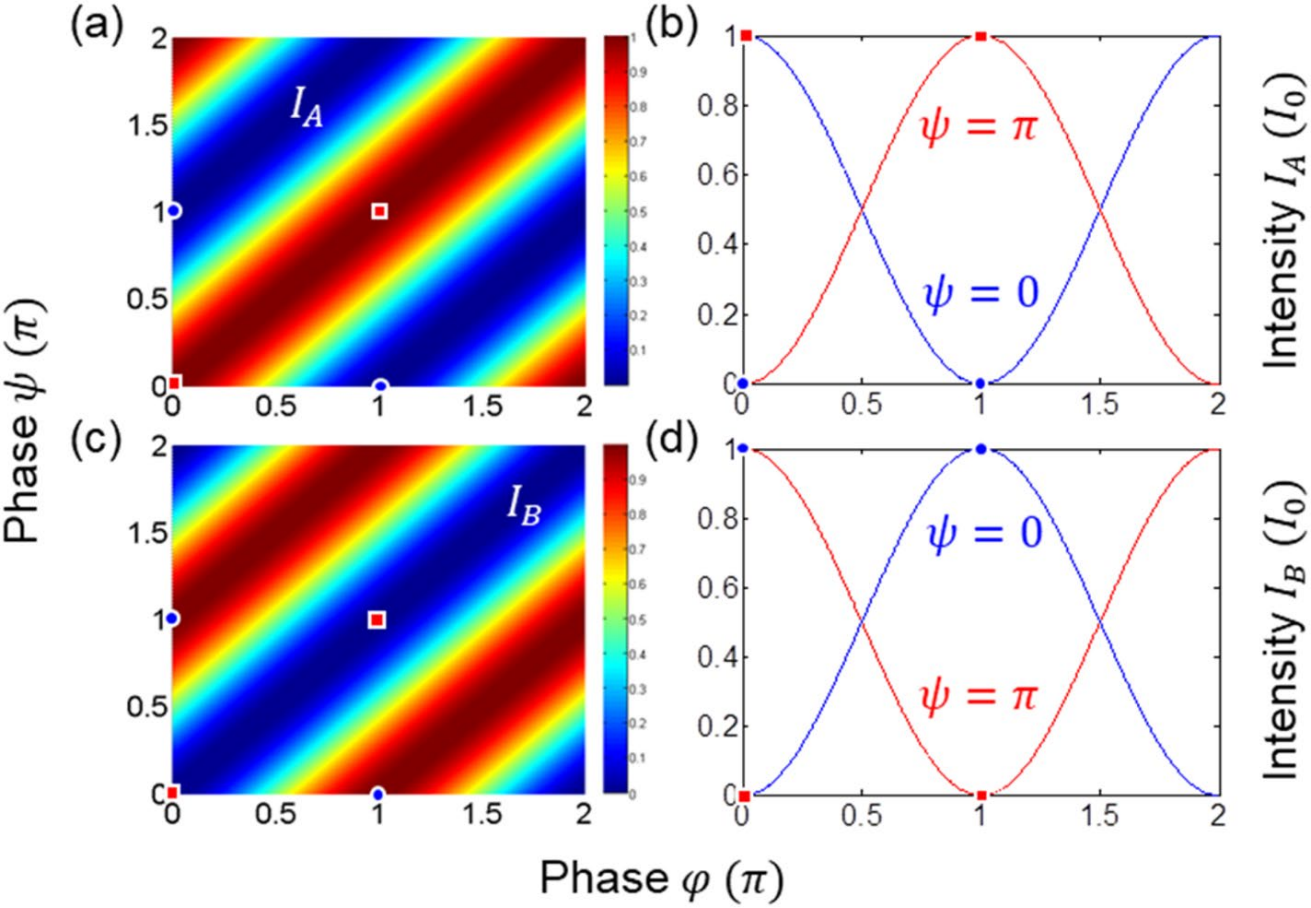
Numerical calculation for Eq. (4). ${{\varphi }} = \varphi_{12}$; ${\uppsi } = \psi_{12}$. (**a**)–(**d**) Red square (blue dot) indicates identity (inversion) relation between two phase bases.

For the case (i), Eq. (4) becomes (see the red square in Fig. 2):

5 $$\left[ {\begin{array}{*{20}c} {E_{A} } \\ {E_{B} } \\ \end{array} } \right] = - e^{i\varphi } \left[ {\begin{array}{*{20}c} 1 & 0 \\ 0 & 1 \\ \end{array} } \right]\left[ {\begin{array}{*{20}c} {E_{0} } \\ 0 \\ \end{array} } \right].$$

Thus, the corresponding intensities are $I_{A} = I_{0}$ and $I_{B} = 0$, resulting in an identity relation: $V_{AB} = 1$,

where the visibility is defined as $V_{AB} = \frac{{V_{A} - V_{B} }}{{V_{A} + V_{B} }}$.

(ii) $$\varphi = 0$$; $${\uppsi } = \pi$$

For the case (ii), Eq. (4) becomes (see the blue dot in Fig. 2):

6 $$\left[ {\begin{array}{*{20}c} {E_{A} } \\ {E_{B} } \\ \end{array} } \right] = ie^{i\varphi } \left[ {\begin{array}{*{20}c} 0 & 1 \\ { - 1} & 0 \\ \end{array} } \right]\left[ {\begin{array}{*{20}c} {E_{0} } \\ 0 \\ \end{array} } \right].$$

Thus, the corresponding intensities are $I_{A} = 0$ and $I_{B} = I_{0}$, resulting in an inversion relation:${ }V_{AB} = - 1$.

(iii) $$\varphi = \pi$$; $${\uppsi } = 0$$

For the case (iii), Eq. (4) becomes (see the blue dot in Fig. 2):

7 $$\left[ {\begin{array}{*{20}c} {E_{A} } \\ {E_{B} } \\ \end{array} } \right] = - ie^{i\varphi } \left[ {\begin{array}{*{20}c} 0 & 1 \\ { - 1} & 0 \\ \end{array} } \right]\left[ {\begin{array}{*{20}c} {E_{0} } \\ 0 \\ \end{array} } \right].$$

Thus, the corresponding intensities are $I_{A} = 0$ and $I_{B} = I_{0}$, resulting in an inversion relation:${ }V_{AB} = - 1$.

(iv) $$\varphi = \pi$$; $${\uppsi } = \pi$$

For the case (iv), Eq. (4) becomes (see the red square in Fig. 2):

8 $$\left[ {\begin{array}{*{20}c} {E_{A} } \\ {E_{B} } \\ \end{array} } \right] = e^{i\varphi } \left[ {\begin{array}{*{20}c} 1 & 0 \\ 0 & 1 \\ \end{array} } \right]\left[ {\begin{array}{*{20}c} {E_{0} } \\ 0 \\ \end{array} } \right].$$

Thus, the corresponding intensities are $I_{A} = I_{0}$ and $I_{B} = 0$, resulting in an identity relation:${ }V_{AB} = 1$.

In a short summary, $I_{A} = I_{0}$ and $I_{B} = 0{ }$ are achieved for ${{\varphi }} = {\uppsi }$, otherwise $I_{A} = 0$ and $I_{B} = I_{0}$. Like Alice’s measurements in Eqs. (2) and (3), Bob also knows Alice’s phase basis choice by measuring his visibility even without communication with her. As a basic property of coherence optics, this propagation directionality in a coupled MZI is the quintessence of USCKD with superposition-caused measurement randomness to an eavesdropper^15^, where the measurement is for the channel attack by an eavesdropper (see section A of the Supplementary Information).

These four options for the key distribution process analyzed in Eqs. (5)–(8) are numerically demonstrated in Fig. 2 by solving Eq. (4). Figure 2a,b are for the output field $I_{A}$, and Fig. 2c,d are for $I_{B}$. Depending on the ${\uppsi } -$ basis choice by Alice given ${{\varphi }} -$ basis chosen by Bob, the visibility $V_{AB}$ becomes either 1 or $- 1$. As an example, for ${{\varphi }} = {\uppi }$ (see the center red squares), Bob surely knows the basis chosen by Alice by his visibility measurements. The key distribution determinacy between Bob and Alice in USCKD is summarized in Table 1.

**Table 1:** Output fields in Fig. 1.

${{\varphi }}$	${{\varphi }}$	${{\varphi }}$
	$0$	${\uppi }$
$0$	${\text{V}}_{AB} = 1$	${\text{V}}_{AB} = - 1$
${\uppi }$	${\text{V}}_{AB} = - 1$	${\text{V}}_{AB} = 1$

$${{\varphi }} = \varphi_{12}$$; $${\uppsi } = \psi_{12}$$. visibility: $$V_{AB} = \frac{{V_{A} - V_{B} }}{{V_{A} + V_{B} }}$$

### Experiments

**Figure 3:**
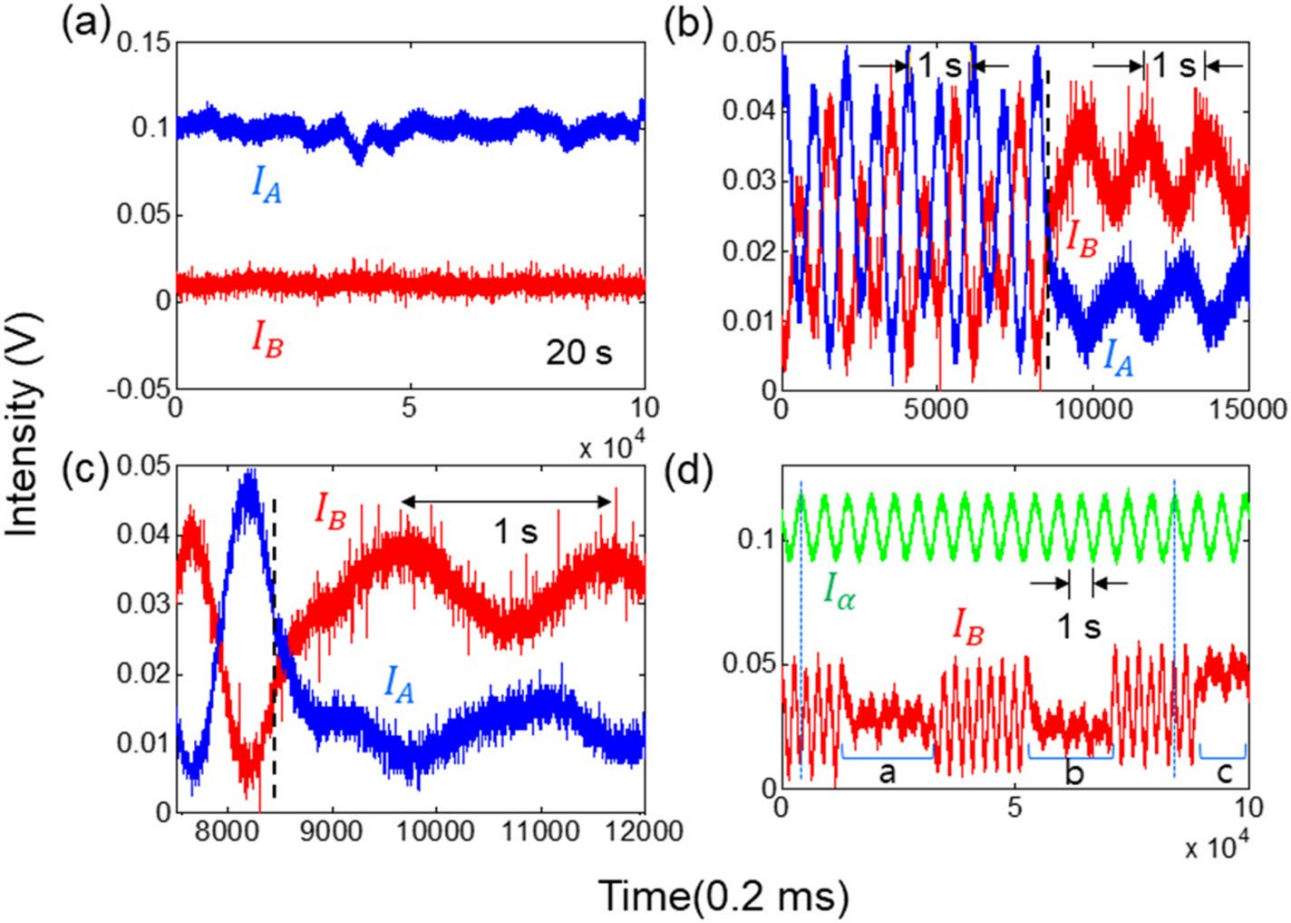
Experimental results for USCKD in Fig. 1. (**a**) $\psi_{12} = \varphi_{12}$. (**b**) Switching between CBW and USCKD. (c) expansion of (**b**). (**d**) Conventional MZI output (green) vs. CBW [USCKD(a/b/c)] (red). In (**b**) and (**c**), $\varphi_{12} = - \psi_{12} = 1 Hz$ before the dashed line; $\varphi_{12} = \psi_{12} = 1 Hz$ after the dashed line. The value of vertical axis is arbitrary.

Figure 3 shows experimental results corresponding to Fig. 2 and Table 1, where four different phase combinations are performed in a cw scheme of the laser light $E_{0}$. The temporal stability is determined mostly by air fluctuations in MZI paths. In Fig. 3, a rough laboratory condition is intentionally applied to the data without any system stabilization, where the MZI stability issue has already been closed^33,34^. Figure 3a shows the MZI channel stability for 20 s for the case of ${\uppsi } = {{\varphi }}$. For this, all four AOMs are set at 80 MHz and ${\uppsi } = {{\varphi }}$. Here, the experimental results of Fig. 3a are the same as in Fig. 2 (see the red squares for ${{\varphi }} = 0$). As mentioned above, the experimental data are from bare laboratory conditions, resulting in ~ 20% phase (path length) fluctuations in short time scales less than a minute. In a long-time scale, the output intensity varies between the minimum and maximum mostly due to air fluctuations.

Figure 3b shows a frequency-dependent phase control of AOM A1 (see Fig. 1). For this, the frequency for A1 is switched to either 1 Hz more or 1 Hz less than AOM A2 at 80 MHz sharp. The other AOMs are set at 80,000,001 Hz for B1 and 80,000,000 Hz for B2, resulting in $\varphi = 1 Hz$ and $\psi = \pm 1 Hz$. The asymmetric structure of the coupled MZIs with ${\uppsi } = - {{\varphi }}$ results in CBW, whose modulation frequency turns out to be doubled (2 Hz), as shown in the region left of the dashed line in Fig. 3b due to $\lambda_{CBW} = \lambda /2$^16^. This doubled frequency is a quantum feature obtained in a classical domain, where it is not the frequency beating. Here, the wavelength ${\uplambda }$ is for the input light $E_{0}$, and $\lambda_{CBW}$ is due to the nonclassical properties $\left( {V_{AB} > 0.71} \right)$ of CBW.

If the symmetric coupling condition is satisfied with ${\uppsi } = {{\varphi }}$, then the identity relation in Eqs. (5) and (8) is satisfied (see both $I_{A}$ and $I_{B}$ in the right region of the dashed line in Fig. 3b), where the nonclassical feature of CBW disappears. The residual 1 Hz (not 2 Hz) modulation is due to the background (leakage) from the first MZI at $\varphi_{12} = 1 Hz$, which is not completely isolated in the experimental setup. Figure 3c is an extension of Fig. 3b, where the output intensities of CBWs are also opposite each other as in the conventional MZI outputs in Fig. 2. Here, the modulation depth $\left( {V_{AB} < 0.71} \right)$ below CBW represents the classical feature of USCKD^35^.

Figure 3d represents toggle switching between CBW and USCKD, where the green curve is for the reference of $I_{\alpha }$ from the first MZI. In the toggle switching by AOM A2 (see the red curve), the intensity value of USCKD depends on the phase of CBW at switching time as denoted in regions ‘a,’ ‘b,’ and ‘c.’ For potential applications of USCKD, such an arbitrary intensity value can be controlled by controlling the internal phase of an rf generator. As already known for CBW bases^35^, USCKD is understood as an extreme of CBW in terms of a symmetric mode in a coupled pendulum model^36^. The alternating CBW peaks between maxima and minima for a fixed value of $I_{\alpha }$ represent the increased phase bases (see the dotted lines). In other words, the ${\uppi }$ span in a single MZI is reduced to ${\uppi }/2$ in the doubly coupled MZI, representing a quantum feature^35^. If an n-coupled MZI is used, then the phase basis span is reduced to ${\uppi }/{\text{n}}$^35^. The related movie is shown in section C of the Supplementary Information for toggle switching between CBW and USCKD.

**Figure 4:**
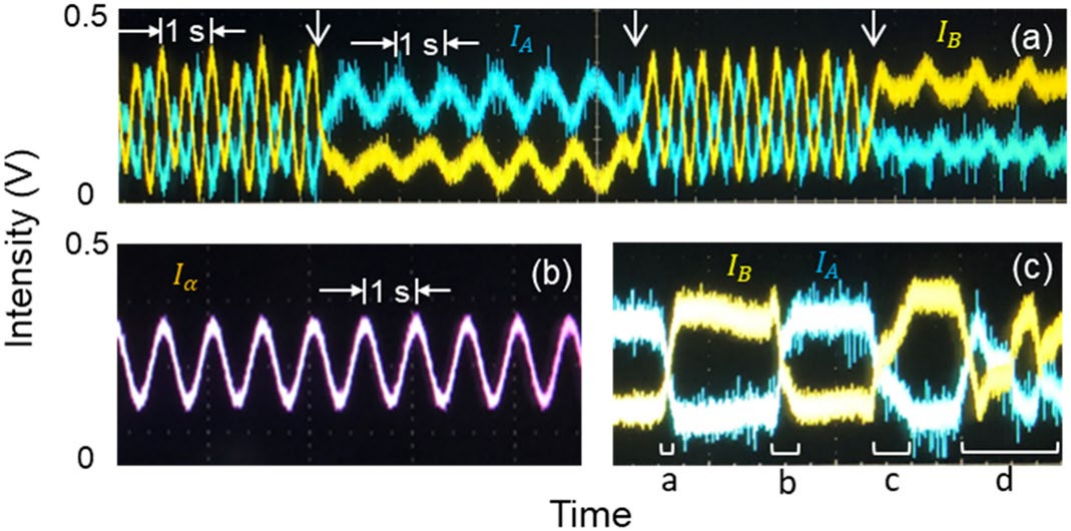
(**a**) CBW vs. USCKD. (**b**) Conventional MZI output $I_{\alpha }$. (**c**) Manual phase control for $\psi_{1}$ in Fig. 1. The driving frequencies for AOM B1 and B2 are 80,000,001 Hz and 80,000,000 Hz. The brackets in (**c**) indicate manual phase $\left( {\uppsi } \right)$ scanning. Intensities in (**a**–**c**) are arbitrary.

Figure 4 shows screen captures of the output intensities from the oscilloscope for Fig. 3. Figure 4a corresponds to Fig. 3b, where both the identity and inversion relations for USCKD in Fig. 2 are shown as results of toggle switching with AOM A2 from CBW. As shown, the maxima and minima of $I_{A}$ and $I_{B}$ are swapped according to a proper phase at the switching time as discussed in Fig. 3(d). Figure 4b is for the $I_{\alpha }$ from the first MZI as a reference, whose modulation frequency (beating) is 1 Hz due to the preset 1 Hz driving frequency difference between AOMs B1 and B2. As mentioned in Fig. 2 as well as Eqs. (5)–(8), Fig. 4c shows ${\uppsi } -$ dependent intensity swapping between $I_{A}$ and $I_{B}$, where the phase control is performed by manually rotating a thin glass inserted into the A1 path of Fig. 1 (see the bracket regions). Here, ${\uppsi } = {{\varphi }} = 0$ is initially set for Fig. 4c. The rotation speed is not constant, but optimistically shows the trend of phase-dependent output-intensity variations. Glass rotation starts at a normal position with respect to the beam path, and thus the phase variation speeds up as it moves from region ‘a’ to ‘d’.

## Discussion

In addition to QKD, experimental demonstration of USCKD provides the proof of principle of unconditional security. As already discussed^15,16^, such unconditional security of USCKD lies in the coupled path superposition between two MZIs, which cannot be obtained conventionlly. Thus, the coupled MZI structure should be differentiated from a single MZI, where the MZI itself belongs to the classical realm. To support the nonclassical property of the coupled MZIs, phase basis-based toggle switching with ${\uppsi } - {{\varphi }} = \pm 1{\text{ Hz}}$ was demonstrated for swapping between CBW and USCKD. Here, the phase bases are orthogonal to each other, representing two modes of the nonclassical features. All aspects of USCKD are macroscopic and coherent. Although the structure of MZIs for USCKD is definitely classical, coupled superposition results in nonclassical features of de Broglie wavelength in an asymmetric form and unitary transformations (identity relation) in a symmetric form. The unitary transformation represents deterministic randomness, where the superposition-caused randomness in MZI is the bedrock of unconditional security in USCKD^15^. Understanding that MZI is another form of BS, where orthogonal input modes are automatically provided^20^, the nonclassical features of USCKD or CBW in the present demonstrations are not trivial. Here, it should be noted that the physical origin of USCKD is the coupled superposition between two MZIs^15,35^.

## Conclusion

Experimental demonstrations of USCKD were presented in a symmetrically coupled MZI structure along with theoretical analyses. The unconditional security of USCKD was provided by deterministic randomness with round trip unitary transformations, where randomness plays a key role for unconditional security via MZI path superposition. The quantum behavior of the coupled MZI structure was confirmed by CBW with coupling manipulations, where the coupled MZIs regenerate fundamental phase bases. For the toggle switching between CBW and USCKD, a $\pm 1{\text{ Hz}}$ frequency difference between the coupled MZIs was used. For the round trip MZI directionality of USCKD, a manual phase $\left( {\uppsi } \right)$ variation with a thin glass was performed, where $0 \le {\uppsi } \le 2{\uppi }$. The MZI stability was tested in bare conditions of MZIs without environmental isolations or a feedback control. Taking advantages of technologically advanced laser locking systems, an active control for MZI phase stability is not an issue anymore, and thus practical applications of USCKD are plausible for fiber-optic communications networks or free space in the future.

## Methods

In Fig. 1, the input light power of $E_{0}$ is around 1 mW, and the diffraction efficiency of AOMs is ~ 70%. The wavelength of $E_{0}$ is 606 nm whose linewidth is ~ 300 kHz, and intensity fluctuation is ~ 1%. The path length of each arm of MZI is ~ 60 cm. All AOM outputs are synchronized by synchronizing rf driving frequency generators, PTS160, PTS250, and AWG3102 (Tektronix) together. Each AOM in the first MZI is without a focused lens pair whose beam diameter is ~ 1 mm. Each AOM in the second MZI, however, is focused and collimated by a 10 cm focal-length lens pair. The fringe pattern of $I_{\alpha }$ and $I_{\beta }$ is a bar shape as usual, while the fringe pattern of $I_{A}$ and $I_{B}$ is an Airy disk type due to the lens-caused circular aperture (see section C of the Supplementary Information). Hamamatsu avalanche photodiodes (C12703) are used to detect the data and recorded on a Tektronix oscilloscope (DPO5204B). For the data in Fig. 3, an iris is added before each detector to pass only the zeroth-order fringe pattern. For the movie in section C of the Supplementary Information, the output light of $I_{B}$ is shined on a paper screen, and the image was captured via iPhone camera. The frequency offset $\pm \delta f$ between two upper paths of the coupled MZI via AOMs is controlled by a two-channel arbitrary function generator (Tektronix AFG3102), whose frequency resolution is 0.001 Hz. All data in Figs. 3 and 4 are raw, single-shot recordings without averaging or trimming. The major error source in data is the air fluctuations in each MZI because the MZI setup of Fig. 1 is uncovered. In other words, the experimental setup is under a rough, coarse, and noisy environment intentionally to show the system’s robustness for potential applications, where phase locking is technologically well matured.

## Supplementary Information

Supplementary Information 1.

Supplementary Information 2.
