# Supplementary material for: Experimental demonstrations of unconditional security in a purely classical regime
Source: Sci Rep. 2021 Feb 18;11:4149. doi: 10.1038/s41598-021-83724-w (PMC7892578; doi:10.1038/s41598-021-83724-w)
Supplement: Supplementary file 2 — Supplementary Information 2. [file 41598_2021_83724_MOESM2_ESM.pdf]

Supplementary Information for

"Experimental demonstrations of unconditional security in a purely classical regime," by

B. S. Ham
GIST, S. Korea

Section A: Eavesdropping randomness in MZI

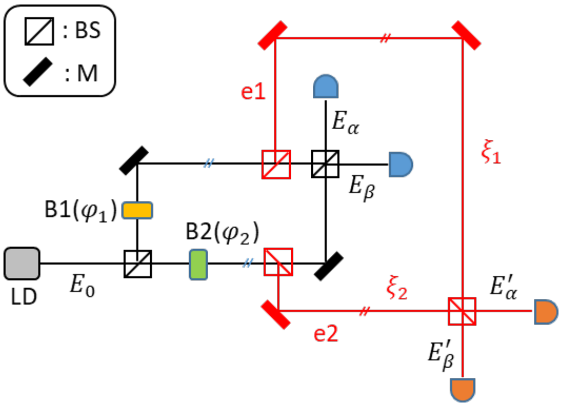

Fig. S1. Schematic of channel attack for Fig. 1. The red lines indicate a channel attack in an MZI. The ξ_j is the phase gain due to the propagation length along the path j.

Figure S1 shows schematic of channel attack by an eavesdropper for Fig. 1. A very sophisticated eavesdropper (Eve) can tap the lines without notice to both parties of Alice and Bob. For Eve, the following interference result is achieved:

$$\begin{bmatrix} E'_\alpha \\ E'_\beta \end{bmatrix} = [BS][\xi][BS]\begin{bmatrix} E_0 \\ 0 \end{bmatrix} = \frac{1}{2}e^{i\xi_2}\begin{bmatrix} 1-e^{i\xi} & i(1+e^{i\xi}) \\ i(1+e^{i\xi}) & -(1-e^{i\xi}) \end{bmatrix}\begin{bmatrix} E_0 \\ 0 \end{bmatrix}, \quad \text{(S1)}$$

where $I'_\alpha = \frac{1}{2}(1-cos\xi)$, $I'_\beta = \frac{1}{2}(1+cos\xi)$, and $\xi = \xi_1 - \xi_2$. Like equation (1) in the main text, equation (S1) also satisfies the directional determinacy between Bob and Eve with the phase ξ. Without doubt, Eve can adjust her interferometer for maxima in visibility, $V_{\alpha\beta}'$: $V_{\alpha\beta}' = \frac{I_\alpha' - I_\beta'}{I_\alpha' + I_\beta'}$. Due to the random phase ξ compared with the original phase φ, however, Eve's measured $V_{\alpha\beta}'$ has a 50% chance of correctness with respect to $V_{\alpha\beta}$ by Alice, resulting in the eavesdropping randomness. Equation (1) also applies to the return paths in the same way.

Section B: Directional determinacy in MZI

According to equation (2) and (3) in the main text, the visibility $V_{\alpha\beta}$ for Alice is shown in Fig. S2. Due to binary phase basis $\varphi \in \{0, \pi\}$, the intensity in each output port has either 0 or 1, resulting in corresponding visibilities at either 1 or -1. This is the directional determinacy in Mach-Zehnder interferometer (MZI) in Fig. 1.

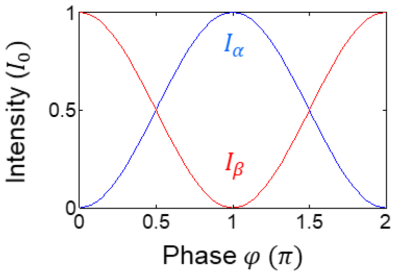

Fig. S2. Numerical calculations for directional determinacy.

Section C: Movie of phase control for CBW and USCKD

Movie 1. $\psi-$dependent switching in the output I_B between CBW and USCKD.
